# Nursing categories’ perceptions of the practice environment and quality of care in North West Province: a cross-sectional survey design

**DOI:** 10.1186/s12912-024-01998-7

**Published:** 2024-06-06

**Authors:** Lufuno M. E. Mphaphuli, Siedine K. Coetzee, Babalwa Tau, Suria M. Ellis

**Affiliations:** 1 NuMIQ Research Focus Area, School of Nursing Science, North-West University, Private Bag X6001, Potchefstroom, South Africa; 2 Unit for Business, Mathematics and Informatics, North-West University, Potchefstroom, South Africa

**Keywords:** Practice environment, Quality of care, Patient safety, Nurses and nurse education

## Abstract

**Background:**

There is a substantial amount of literature on the perception of the practice environment and quality of care as perceived by registered nurses and community services nurses in South Africa and worldwide, but there is little to no research that could be found regarding other categories of nurses, and how these perceptions differ between the different categories. Therefore, the aim of this study is to describe the different nursing categories’ perceptions of the practice environment and quality of care and the association between the variables.

**Methods:**

This study applied a cross-sectional survey design. Data were collected in April 2021 in the public sector of the North West Province. Multiphase sampling was applied to all categories of nurses who worked in an in-patient unit in the selected hospital for at least 3 months (*n* = 236).

**Results:**

All nursing categories perceived the practice environment as negative, regarding nurse participation in hospital affairs; nurse manager ability, leadership, and support of nurses and staffing and resource adequacy. Perceived quality of care and patient safety items were perceived as neutral and good. However, in all instances, the perceptions of community service nurses and registered nurses were most negative, and enrolled nurse assistants most positive. Adverse events towards patients and nurses were perceived to only occur a few times a year. Overall, nurse perceptions of quality of care and patient safety were most correlated with the subscale of nurse foundations of quality of care and nurse manager ability, leadership, and support of nurses. Adverse events towards patients were most correlated with the collegial nurse-physician relationship subscale, while adverse events towards nurses were correlated with the foundations of quality of care subscale.

**Conclusion:**

Improving the practice environment, especially regarding the subscale nurse foundations of quality of care and nurse manager ability, leadership, and support of nurses, is associated with improved quality of care. Nurses with higher qualifications, registered nurses and community service nurses rated quality of care lower than other categories of nurses, contributing to literature that higher qualified staff are more competent to assess the practice environment and quality of care.

## Background

The practice environment is defined as “the organizational characteristics of a work setting that facilitate or constrain professional nursing practice” [1]. The practice environment has been studied for decades in different countries and settings, and has been recognised as the single most important aspect influencing nurse and patient outcomes [2]. The practice environment has been shown to positively influence nurse empowerment [3], job satisfaction [4], organisational commitment [5], clinical autonomy and control over practice [6]. Conversely, it was negatively associated with burnout [7], job dissatisfaction and intent to leave [8]. With regard to patient outcomes, the practice environment was positively associated with improved nurse-rated quality of care [4], quality end-of-life care [9], patient and family satisfaction [10], patient safety and self-care ability [11]. Furthermore, it was negatively associated with postoperative complications, failure to rescue and mortality [12], nosocomial infections, patient falls, catheter line sepsis, and medication errors [13].

Quality of care is defined as “the degree to which health care services for individuals and populations increase the likelihood of desired health outcomes and are consistent with current professional knowledge” [14]. Quality of care consists of six domains, namely safety, effectiveness, patient- centeredness, timeliness, efficiency and equitableness [14]. Richardson et al. [14] describe patient safety as an integrated dimension of quality of care, and thus nurse-reported patient safety was also assessed in this study. Gqaleni and Bhengu [15] defined patient safety as a focus on the prevention of errors that are made in healthcare to ensure that no harm befalls the patient.

Nurses are at the forefront of patient care delivery. They work with patients 24/7 and are directly involved in all facets of patient care; thus they play a critical role in the assessment of quality of care and patient safety [2]. In fact, McHugh and Stimpfel [16] state that nurse-rated quality of care is a valuable indicator of hospital performance. Research has specifically shown that higher nurse-rated quality of care was associated with lower odds of both mortality and failure to rescue; higher composite process-of-care scores for acute myocardial infarction, pneumonia, and surgical patients; improved evaluations of hospital care experience by patients; and patients recommending the hospital [16]. Tvedt et al. [11]. found that not only was nurse-rated quality of care associated with increased survival, it was also associated with nurse assessment of staffing adequacy, exemplifying that nurses’ characterisation of the microsystem in which they work truly reflects the general performance of the hospital. Thus Pinder et al. [17]. suggest that nurses’ feedback may be useful in assessing the overall hospital performance, as well as in raising questions and seeking answers on how negative feedback could be redirected towards a positive path.

Research conducted on the association between the practice environment and the nurse-rated quality of care and patient safety showed that hospitals with a positive practice environment were associated with lower rates of mortality and failure to rescue [18, 19], fewer readmissions to hospital (within 30 days) [20], less hospital-acquired pressure ulcers [21], less information being lost during shift changes, fewer nursing care tasks being left undone [22], nurses expressing increased confidence that patients were ready for discharge [2, 18, 23], nurse-reported fair/poor ward quality, and nurse-reported poor/failing safety grade [2].

While there was already a substantial amount of literature on the perceptions of the practice environment and quality of care of registered nurses (RNs), and community service nurses (CSNs - new graduate nurses) in South Africa and worldwide [2, 24–27], there was little to no research that could be found with regard to other categories of nurses, and how these perceptions differ between the different categories.

Globally there are different categories of nurses, and in South Africa they are classified as follows: RN (4-year degree or 4-year diploma), with a sub-category of a CSN (a new graduate nurse), enrolled nurse (EN) (2-year diploma) and the enrolled nurse assistant (ENA) (1-year certificate). This is similar to the system in the United States of America or in Europe and Australia, that has a Bachelor’s Degree in Nursing (4 years), an Associate Degree or Diploma in Nursing (2 years) and a Certificate in Nursing (1 year). Nurses with a 2-year associate degree or diploma work under the supervision of the RN and can provide basic nursing care. Nurses with a 1-year certificate work under the direction of the RN and are responsible for elementary nursing care. Therefore, the perceptions of the practice environment and quality of care of nurses with a 2- year associate degree or diploma and those with a 1-year certificate are just as important, as they do much of the bedside nursing.

With a focus on education, research has shown that hospitals with better-qualified workforces have better patient outcomes [28], that increased levels of education are associated with decreased perceptions of quality of care and patient safety, possibly due to increased awareness and more accurate evaluation of the practice environment and the care that is rendered [29]. Very few studies have researched direct comparisons between different categories of nurses and their perceptions of the practice environment and quality of care in their units.

Regionally, Alhassan et al. [30]. , found that RNs had a greater knowledge and awareness of adverse medical events than the ENAs did. In South Africa, a study by Blignaut et al. [31]. , found that RNs with degree and diploma qualifications had similar results with regard to perceptions of quality of care and patient safety, and that they rated these aspects in their unit similarly. Swart et al. [32]. found that ENs rated error prevention strategies more favourably than RNs did, but also rated losing patient information during shift changes and patient transfers, and mistakes being held against staff more negatively than did RNs.

From this background it becomes clear that there is a substantial amount of literature on RNs’ and CSNs’ perceptions of the practice environment and quality of care; however, there was limited and almost no information about ENs’ and ENAs’ perceptions regarding these variables. The few studies that had conducted direct comparisons between especially RNs and ENs appeared to have contrasting results. Therefore, this study aims to describe the different nursing categories’ perceptions of the practice environment and quality of care, and the association between the variables.

## Theoretical framework

In this study the researcher subscribes to the modification of Donabedian [33] and Battles’ [34] structure, process and outcome model as described by Tvedt et al. [35]. .

In this model, the focus is on achieving good patient outcomes (inner circle), which is influenced by the hospital structure (outer circle) and work environment (also practice environment) processes (middle circle). Good patient outcomes are determined by four factors in this model, namely quality of nursing care, patient safety, low frequency of adverse events and patient self-care ability. These will all be measured in this study. Practice environment processes focus on the quality of the system; patient safety management; nurse manager ability, leadership and support of nurses; staffing and resource adequacy; collegial nurse-physician relations; nurse participation in hospital affairs; nursing foundations of quality of care; and education and career. These will also be measured in this study, with a focus on the association between the practice environment and quality of care according to the perceptions of the different nursing categories.

## Methods

This study applied a cross-sectional survey design in the North West Province (NWP) of South Africa (SA). The NWP is divided into four district municipalities, which are further subdivided into 18 local municipalities. There are 23 public hospitals in the NWP. SA has a dual healthcare system, namely a public sector that provides care to approximately 83% of the population, and a private sector that provides care to the rest of the population. In the public sector a financial means test is applied to determine whether patients qualify for access to free healthcare services, otherwise they pay a small fee corresponding with their income; in the private sector healthcare is paid by a medical scheme or out of pocket [36]. This study is conducted in the public healthcare sector, which has four hospital levels, namely central; tertiary, regional and district hospitals, which offer different healthcare services, ranging from preventative to curative.

### Instrumentation

This study used a valid and reliable self-report, paper-based survey that measures four variables: the Practice Environment Scale of the Nurse Work Index (PES-NWI), quality of care, patient safety, adverse events and selected nurse and unit demographics.

#### Practice Environment Scale of the nurse work index (PES-NWI)

The PES-NWI was used to measure the practice environment of the nurse. It consists of five subscales, namely nurse participation in hospital affairs; nursing foundations for quality of care; nurse manager ability, leadership and support of nurses; staffing and resource adequacy; and the collegial nurse-physician relations. This scale consists of 32 questions answered on a Likert scale ranging from 1 to 4, where 1 represents strongly disagree and 4 strongly agree; a mean score of 2.5 or more indicates a positive practice environment. Scores on the PES-NWI are valid and reliable for measuring the nursing practice environment across samples in the United States of America and elsewhere [37].

#### Quality of care

Nurse-perceived quality of care was measured using 4 questions that are used as single items. These questions have been used in multi-country studies in Europe [38], North America [39], Asia [40], and SA [25], and have been included in several systematic reviews and meta-analyses [2]. These questions were answered on a Likert scale from 1 to 4, where 1 represents frequently and 4 represents never.

#### Patient safety

Two questions measuring nurse-perceived patient safety, that have been used as single items in multi-country studies in Europe [38], North America [39], Asia [40], and SA [25], and have been included in several systematic reviews and meta-analyses [2] were used in this study. These questions were answered on a Likert scale from one to five, where one represents excellent and five represents failing. The other eight items came from the Agency for Healthcare Research and Quality (AHRQ) surveys on patient safety (SOPS) culture [41]. They were answered on a Likert scale from one to five, where one represents strongly agree and five strongly disagree.

#### Adverse events

Nurse-perceived adverse events consisted of 7 questions, that have been used as single items in multi-country studies in Europe [38], North America [39], Asia [40], and SA [25], and have been included in several systematic reviews and meta-analyses [2]. These questions were answered on a Likert scale from 0 to 5, where 0 represents never and 5 represents daily.

#### Demographics

Selected personal and unit demographics were also included in the study, which included age, gender, employment status, nursing category, Bachelor’s degree in Nursing, specialty of current unit, clinical specialty in nursing, years worked as a nurse, and years worked in the hospital.

### Population and sampling

A multiphase sampling method was applied in this study. NW was purposively selected as the province of choice for this study. The public sector was purposely chosen. Within the public sector, purposive sampling was applied to the selection of the hospitals, namely the largest tertiary hospital in the province and the closest surrounding regional and district hospitals (*n* = 3). There is no central hospital in this province. Total population sampling according to Lund [42] was applied when selecting the units and participants for the study. All in-patient units, including the emergency department and theatre were included in the study. The participants consisted of registered nurses and midwives, CSNs, ENs and ENAs. Inclusion criteria that were applied is that nurses must have worked in the selected hospitals for at least 3 months, since persons with less than 3 months may not be able to give an overall assessment on aspects such as the practice environment, quality of care or patient safety within their units or even hospitals, as they are still new employees. At the tertiary hospital *N* = 319 surveys were distributed and 123 (n) were returned, at the regional hospital *N* = 146 surveys were distributed and 72 (n) returned, finally in the small district hospital had *N* = 76 had been distributed and 41 (n) returned. The total sample consisted of *n* = 236 nurses, with a response rate of 43.6%.

### Data collection

Data was collected in April 2021. The Chief Executive Officers served as gatekeepers, and referred the researcher to the nursing service managers of the facilities, who acted as mediators in the study. The mediator informed unit managers of the research study during their weekly meeting, and the researchers was also available to attend this meeting (either in person or online) to answer any questions or queries. Thereafter, the unit managers informed the nursing personnel of the research study, and when to expect the researcher.

The researcher presented an overview of the research project and the details of the informed consent form to the potential participants to consider participation in the study, in the presence of an independent person. Thereafter the potential participants were provided with an informed consent form, which was left in their possession for 24 h. The independent person obtained the written informed consent from nursing staff who were willing to participate in the study with observance to COVID-19 protocols. Once signed by all parties, the informed consent form was sealed in a DL envelope and kept safely with the independent person. Upon receipt of the informed consent form, the independent person provided each participant with a survey, an NWU-branded pen and a sachet of coffee as tokens of appreciation, in a large unsealed C4 envelope, which could be completed anonymously at any time and place most convenient for the participant. A separate leaflet explaining entry into the lucky draw was included in the envelope. The participant had two days to complete the survey. After the participants completed the survey, they sealed it in the C4 envelope provided and posted it in a box with a post-split in the mediator’s office or staff commons. After one week, the researcher collected all the boxes.

### Data analysis

The paper-based surveys were captured with an optical mark recognition (OMR) scanner, and imported on a Microsoft Excel Spreadsheet. Hospitals and units were anonymised. Descriptive statistics were used to profile the characteristics of nurse participants and units of the selected hospitals. Confirmatory and exploratory factor analysis and Cronbach’s Alpha were conducted to ensure the validity and reliability of the scales. Missing data required for calculating the factor scores (dependent variables) were handled by mean replacement. Analysis of variance (ANOVA) were conducted to determine the associations between practice environment, quality of care, patient safety and adverse events which were perceived by the different categories of nurses. Cohen’s d was used as an effect size measure to determine the importance of the differences in means, where 0.2 is small; 0.5 medium and 0.8 large, respectively. While Spearman rank order correlations were used to determine the association between the practice environment, quality of care, patient safety and adverse events. The magnitude of correlations is regarded as the effect size, where 0.1 is small; 0.3 medium and 0.5 large.

### Ethics

This study received ethical approval from North-West University Health Research Ethics Committee (NWU-00269-21-A1) and the NWP Department of Health (DoH). The CEOs of each of the hospitals that were part of the study were asked for their goodwill permission. Consent was acquired from every nurse manager to enter the unit. Written informed consent was obtained from every participant. The completion of surveys was anonymous. Ethical considerations for this study were in line with the ethical codes and standards [43].

## Results

**Table 1:** Personal and unit demographics (*n* = 236)

Personal Demographics			
Variables	Categories	Frequency (*n*)	Percentage (%)
Gender	Male Female	43 193	18.22 81.78
Employment status	Permanently employed (full-time) Permanently employed (part-time) Agency/temporarily employed Missing	202 8 25 1	85.59 3.39 10.59 0.42
Nursing category	RN and/or midwife CSN EN and/or midwife ENAs Missing	135 7 31 62 1	57.20 2.97 13.14 26.27 0.42
Bachelor’s degree in Nursing	Yes No	46 96	32.39 67.61
Specialty of current unit	Medical Surgical Trauma Maternity Critical Care Theatre Paediatrics Other Missing	47 21 24 37 27 14 20 45 1	19.90 8.90 10.20 15.70 11.40 5.90 8.50 19.10 0.4
Clinical specialty in nursing	Yes No Missing	51 182 3	21.60 77.10 1.3
Hospitals	Public Hospital 1 Public Hospital 2 Public Hospital 3	123 72 41	52.12 30.51 17.37
	**Range**	**M**	**SD**
Age (yrs.)	22–62	40.41	10.07
Years worked as a nurse	0–39	12.57	10.01
Years worked in hospital	0–39	9.45	8.85

Table 1 represents the personal and unit demographic details of the participants. The total number of participants in the NWP public hospitals was 236 (n). The majority of participants in the study were female (81.78%) and employed full time (85.59%). RNs and/or midwives made up the largest category of participants (57.20%), of whom 32.39% had a Bachelor’s degree in nursing. Most participants worked in medical (19.90%), maternity (15.70%) and critical care (11.40%) units.

Only 21.90% (*n* = 51) had specialty training in a field of nursing.The descriptive statistics indicated that the average age was 40.41 years (SD = 10.07). Most of the participants had been working as a nurse for approximately 12.57 years (SD = 10.01) and for approximately 9.45 years in the current hospital (SD = 8.85).

**Table 2:** Descriptive statistics and reliability of main study variables

**Practice Environment**				
Variables	Score range	Mean	SD	Reliability
Nurse participation in hospital affairs	1–4	2.29	0.70	0.88
Nurse foundations of quality of care	1–4	2.88	0.58	0.84
Nurse manager ability, leadership and support of nurses	1–4	2.45	0.79	0.84
Staffing and resource adequacy	1–4	2.34	0.75	0.75
Collegial nurse-physician relationship	1–4	2.86	0.71	0.89
**Patient Safety**				
Please give your current practice setting an overall grade on patient safety? Communication errors Relies too much on temporary or agency staff Staff feel like their mistakes are held against them There is a lack of support for staff involved in patient safety errors	1–5 1–5 1–5 1–5 1–5	2.23 2.34 3.28 2.53 2.71	1.01 0.85 1.42 1.27 1.32	Single item 0.75 Single item Single item Single item
**Adverse Events**				
Adverse events affecting patients	1–5	1.71	0.70	0.79
Adverse events impacting nurses	1–5	2.20	0.87	0.82
**Quality of Care**				
In general, how would you describe the quality of nursing care delivered to patients in your work setting? Would you recommend your place of work to your family and friends needing healthcare? How sure are you that management will act to resolve problems in patient care that nurses identify? How sure are you that your patients and their caregivers can manage their care after discharge?	1–4 1–4 1–4 1–4	2.12 2.02 2.5 2.52	0.78 0.96 0.99 0.88	Single item Single item Single item Single item

The PES-NWI was considered reliable, with all Cronbach’s alphas ranging from 0.75 to 0.89 (see Table 2). The nurses perceived the practice environment as positive regarding nurse foundations of quality of care (M = 2.88; SD = 0.58) and collegial nurse-physician relationship (M = 2.86; SD = 0.71), while the other three subscales were experienced as unfavourable.

With regard to nurse-perceived patient safety, an exploratory factor analysis was conducted with the AHRQ items, and three factors emerged. However, only one factor was considered reliable, namely communication errors (a = 0.75), that consisted of the following five items: regularly review work processes to determine if changes are needed to improve patient safety; staff speak up if they see something that may negatively affect patient care; we discuss ways to prevent errors from happening again; staff feel free to question the decisions or actions of those in authority; and the actions of hospital management show that patient safety is a top priority. All the other AHRQ SOPS items were analysed as single items.

According to the nurse-perceived quality of care items, nurses described the quality of nursing care delivered to patients in their work setting as good (M = 2.12; SD = 0.78); they further indicated that they would probably recommend their place of work to their family and friends needing healthcare (M = 2.02; SD = 0.96). However, nurses were only somewhat confident that management will act to resolve problems in patient care that nurses identify (M = 2.5; SD = 0.99) and that patients and their caregivers could manage their care after discharge (M = 2.52; SD = 0.88).

Nurses felt that the current practice had a good overall grade on patient safety (M = 2.23, SD = 1.01), but felt neutral regarding communication to prevent errors (M = 2.34; SD = 0.85). They were in disagreement with the fact that the hospital relies too much on temporary or agency staff (M = 3.28; SD = 1.42) but remained neutral when it came to their mistakes being held against them (M = 2.53; SD = 1.27), and a lack of support for staff involved in patient safety errors (M = 2.71; SD = 1.32).

An exploratory factor analysis was conducted with the Adverse Event (AE) data, and two factors emerged adverse events affecting patients (medication errors; patients fall with injury after admission; healthcare-associated infections and treatments/procedures resulting in unintended harm) and adverse events affecting nurses (work-related physical injuries; complaints from patients/their families; both physical and verbal abuse from patients/their families; and needlestick injuries). Nurse perceived adverse events that impact on patients as occurring a few times in a year (M = 1.71; SD = 0.70). Likewise, adverse events that impacted nurses were also perceived as occurring a few times a year (M = 2.20; SD = 0.87).

**Table 3:** Effect sizes between nurse categories, and the practice environment, quality of care and patient safety

PES-NWI	Nurse category	*n*	Mean	Std. Deviation	*P* (Statistical Significance)	d (Effect Size) RN and/ or midwife	d CSN	d EN and/or midwife	Lower band 95% CI (Confidence Interval)	Upper band95% CI
Nurse manager ability, leadership and support of nurses	RN and/or midwife	129	2.36	0.81	0.031*				2.22	2.50
	CSN	6	2.36	0.71		0.00			1.79	2.93
	EN and/or midwife	30	2.39	0.75		0.04	0.04		2.12	2.66
	ENA	57	2.72	0.73		0.45	0.50	0.44	2.53	2.91
Staffing and resource adequacy	RN and/or midwife	130	2.27	0.74	0.011*				2.14	2.39
	CSN	6	1.88	0.79		0.50			1.25	2.50
	EN and/or midwife	30	2.31	0.71		0.05	0.55		2.05	2.56
	ENA	57	2.61	0.73		0.47	0.94	0.42	2.42	2.80
**Patient Safety**	**Nurse Category**	**n**	**Mean**	**Std. Deviation**	**P (sig)**	**d** **Registered nurse and/and or midwife**	**d** **Community Service Nurse**	**d** **Enrolled nurse and/or midwife**	**Lower band 95% CI**	**Upper band 95% CI**
Please give your current practice setting an overall grade on patient safety?	RN and/or midwife	131	2.40	0.967	<0.001***				2.24	2.57
	CSN	7	3.00	1.155		0.52			2.14	3.86
	EN and/or midwife	29	2.03	0.906		0.38	0.84		1.70	2.36
	ENA	61	1.85	0.997		0.55	0.99	0.18	1.60	2.10
**Quality of Care**	**Nurse Category**	**n**	**Mean**	**Std. Deviation**	**P (sig)**	**d** **Registered nurse and/and or midwife**	**d** **Community Service Nurse**	**d** **Enrolled nurse and/or midwife**	**Lower band 95% CI**	**Upper band 95% CI**
In general, how would you describe the quality of nursing care delivered to patients in your work setting?	RN and/or midwife	131	2.21	0.755	<0.001***				2.08	2.34
	CSN	7	3.14	0.378		1.23			2.86	3.42
	EN and/or midwife	29	2.10	0.772		0.14	1.35		1.82	2.38
	ENA	61	1.80	0.726		0.54	1.84	0.39	1.62	1.99
Would you recommend your place of work to your family and friends needing healthcare?	RN and/or midwife	131	2.06	0.892	<0.001***				1.91	2.21
	CSN	7	3.71	0.488		1.85			3.35	4.08
	EN and/or midwife	29	2.00	0.926		0.07	1.85		1.66	2.34
	ENA	61	1.74	0.947		0.34	2.09	0.28	1.50	1.98
How confident are you that management will act to resolve problems in patient care that nurses identify	RN and/or midwife	130	2.76	0.930	<0.001***				2.60	2.92
	CSN	7	3.43	0.535		0.72			3.03	3.82
	EN and/or midwife	29	2.76	0.872		0.00	0.77		2.44	3.08
	ENA	61	2.07	1.031		0.68	1.32	0.67	1.81	2.32
How confident are you that your patients and their caregivers can manage their care after discharge?	RN and/or midwife	130	2.61	0.831	0.005**				2.46	2.75
	CSN	7	3.29	0.756		0.82			2.73	3.85
	EN and/or midwife	29	2.52	0.911		0.10	0.84		2.19	2.85
	ENA	61	2.25	0.907		0.40	1.15	0.30	2.02	2.47

*Note* *p* (Statistical Significance) ***<0.001, **<0.01, *<0.05

d (Effect Size) 0.2 = small, 0.5 = medium, 0.8 = large

*RN* = Registered Nurse; *CSN* = Community Service Nurse; *EN* = Enrolled Nurse; *ENA* = Enrolled Nurse Assistant

Only statistically significant associations are reported on (see Table 3). There was a medium effect size (d = 0.44–0.50; *p* = 0.031*) found with perception of nurse manager ability, leadership and support of nurses, where ENAs experienced this subscale more positively (M = 2.72) than any of the other categories of nurses (M = 2.36 [RNs/midwives]; M = 2.36 [CSNs]; M = 2.39 [ENs/midwives]). There was a medium to large effect size (d = 0.42–0.94; *p* = 0.011*) found with staffing and resource adequacy, where ENAs experienced this subscale more positively (M = 2.61) than RNs (M = 2.27), CSNs (M = 1.88) and ENs (M = 2.31); CSNs had a medium effect size (d = 0.50; *p* = 0.011*) and experienced this subscale more negatively than all of the other categories of staff.

Regarding giving the unit an overall grade on patient safety, CSNs had a medium to large effect size (d = 0.52–0.99; *p* = <0.001***) rated this aspect more negatively (M = 3.00) than RNs (M = 2.40), ENs (M = 2.03) and ENAs (M = 1.85), and there was a medium effect (d = 0.38–0.55; *p* = <0.001***) between RNs and other categories of staff, with RNs rating this aspect more negatively. There were no statistically significant associations with adverse events.

There were some statistically significant relationships between nurse categories and nurse- perceived quality of care items. In describing the quality of nursing care delivered to patients in the work setting, there were large effect sizes (d = 1.23–1.84; *p* = <0.001***) between CSNs (M = 3.14), that experienced this more negatively that all other categories of staff (RNs M = 2.21; ENs M = 2.10; ENAs M = 1.80), while ENAs had a medium effect size (d = 0.39–0.54; *p* = <0.001***) and experienced this aspect more positively than all other categories of staff. Regarding recommending your place of work to family and friends needing healthcare, there was a large effect (d = 1.85–2.09; *p* = <0.001***) between CSNs, who rated this aspect much poorer than all other categories of staff (RNs M = 2.06; ENs M = 2.00; ENAs M = 1.74), and ENAs had a small effect size (d = 0.28–0.34; *p* = <0.001***), experiencing this aspect slightly more positively that other categories of staff. Regarding confidence in management to act in resolving problems in patient care that nurses identify, there were large effects between both the ENAs (d = 0.67–1.32; *p* = <0.001***) who rated the aspect more positively (M = 2.07), and CSNs (d = 0.72–1.32; *p* = <0.001***) who rated this aspect more negatively (M = 3.43), and all other categories of staff (RNs M = 2.76; ENs M = 2.76). Finally, regarding how confident they are that patients and caregivers can manage their care after discharge, CSNs has a large effect size (d = 0.82–1.15; *p* = <0.001***) and experienced this aspect more negatively than RNs (M = 2.61); Ens (M = 2.52) and ENAs (M = 2.25), while ENAs had a medium effect (d = 0.30–0.40; *p* = <0.001***), experiencing this aspect more positively that other categories of staff.

**Table 4:** Correlations between the practice environment, quality of care and patient safety

		PES-NWI – Nurse participation in hospital affairs	PES_NWI_ Nurse foundations of quality of care	PES_NWI_ Nurse Manager Ability Leadership and Support Nurse	PES_NWI_ Staffing and Resources Adequacy	PES_NWI_ Collegial Nurse-Physician Relation
**Patient Safety**						
Please give your current practice setting an overall grade on patient safety	Correlation coefficient Sig. (2tailed) N	-0.291*** <0.001 221	-0.479*** <0.001 221	-0.355*** <0.001 221	-0.309*** <0.001 221	-0.327*** <0.001 221
Communication errors	Correlation coefficient Sig. (2-tailed) N	-0.400*** <0.001 217	-0.454*** <0.001 217	-0.467*** <0.001 217	-0.300*** <0.001 217	-0.274*** <0.001 217
Relies too much on temporary or agency staff	Correlation coefficient Sig. (2-tailed) N	0.117 0.084 218	0.227** 0.001 218	0.138* 0.042 218	0.080 0.237 218	0.082 0.230 218
Staff feel like their mistakes are held against them	Correlation coefficient Sig. (2-tailed) N	0.297*** <0.001 216	0.055*** <0.001 216	0.320*** <0.001 216	0.149* 0.028 216	0.160* 0.018 216
There is a lack of support for staff involved in patient safety errors	Correlation coefficient Sig. (2-tailed) N	0.255*** <0.001 214	0.298*** <0.001 214	0.342*** <0.001 214	0.145* 0.034 214	0.203** 0.003 214
**Adverse events**						
Adverse events affecting patients	Correlation coefficient Sig. (2-tailed) N	-0.100 0.144 216	− 0.266*** <0.001 216	-0.116 0.090 216	− 0.187** 0.006 216	− 0.301*** <0.001 216
Adverse events impacting nurses	Correlation coefficient Sig. (2-tailed) N	-0.165* 0.015 216	-0.283*** <0.001 216	-0.197** 0.004 216	-0.180** 0.008 216	-0.208** 0.002 216
**Quality of care**						
In general how would you describe the quality of nursing care delivered to patients in your work setting?	Correlation coefficient Sig. (2-tailed) N	-0.295*** <0.001 221	-0.490*** <0.001 221	-0.348*** <0.001 221	-0.387*** <0.001 221	− 0.371*** <0.001 221
Would you recommend your place of work to your family and friends needing healthcare?	Correlation coefficient Sig. (2-tailed) N	-0.308*** <0.001 221	-0.442*** <0.001 221	-0.385*** <0.001 221	-0.298*** <0.001 221	-0.317*** <0.001 221
How sure are you that management will act to resolve problems in patient care that nurses identify?	Correlation coefficient Sig. (2-tailed) N	-0.486*** <0.001 220	-0.475*** <0.001 220	-0.498*** <0.001 220	-0.433*** <0.001 220	-0.343*** <0.001 220
How confident are you that your patients and their caregivers can manage their care after discharge?	Correlation coefficient Sig. (2-tailed) N	-0.222** 0.001 220	-0.409*** <0.001 220	-0.288*** <0.001 220	-0.289*** <0.001 220	-0.245*** <0.001 220

According to Table 4, the correlations between the practice environment subscales and nurse-perceived quality of care indicated that the subscale of nurses foundations of quality of care had the highest correlations with the quality of nursing care delivered to patients in their work setting (*r*=-0.490***; *p* = <0.001), as well as recommending their place of work to their family and friends needing healthcare (*r*=-0.442***; *p* =< 0.001) and confidence that patients and their caregivers can manage their care after discharge (*r*=- 0.409***; *p* = <0.001). The nurse manager ability, leadership and support of nurses’ subscale had the largest correlation with nurses being sure that management will act to resolve problems in patient care that they have identified (*r*=-0.498***; *p* = <0.001).

Patient safety had the highest correlations with nurse manager ability, leadership and support of nurses (communication errors [*r*=-0.467***; *p* = <0.001], staff feeling like their mistakes are held against them [*r* = 0.320***; *p* = <0.001] and lack of support for staff involved in patient safety errors [*r* = 0.342***; *p* = <0.001]. The subscale of nurse foundations of quality of care was most highly correlated with overall grade on patient safety (*r*=-0.479***; *p* = <0.001) and relies too much on temporary or agency staff (*r* = 0.227; *p* = 0.001).

The collegial nurse-physician relationship subscale had the largest negative correlation with adverse events affecting patients (*r*=-0.301***; *p* = <0.001) and the foundations of quality of care subscale had the largest negative correlation with adverse events impacting nurses (*r*=-0.283***; *p* = <0.001).

## Discussion

All categories of nurses perceived the practice environment as positive in terms of nurse foundations of quality of care and collegial nurse-physician relationship, but experienced nurse participation in hospital affairs; nurse manager ability, leadership and support of nurses; and staffing and resource adequacy negatively. According to the nurse-perceived quality of care items, all categories of nurses described the quality of nursing care delivered to patients in their work setting as good, and they further indicated that they would probably recommend their place of work to their family and friends needing healthcare. However, all categories of nurses were only somewhat confident that management will act to resolve problems in patient care that nurses identify or that patients and their caregivers could manage their care after discharge. All categories of nurses felt neutral regarding communication errors and that the hospital relies too much on temporary or agency staff. They remained neutral when it came to their mistakes being held against them, and a lack of support for staff involved in patient safety errors. Nurses perceived adverse events which affected patients or nurses as occurring only a few times a year.

In all instances, the perceptions of CSNs were most negative, however, it must be stated that it is a small sample, and therefore these findings have limited generalizability. This was then followed by the RNs and ENs, while ENAs always had the most positive perceptions. Nurse perceptions of quality of care and patient safety were most correlated with the subscale of nurse’s foundations of quality of care and nurse manager ability, leadership and support of nurses, while adverse events affecting patients was most correlated with the collegial nurse-physician relationship subscale and adverse events impacting nurses with the foundations of quality care subscale.

Nurses (all categories) were least satisfied with the practice environment with regard to nurse participation in hospital affairs; nurse manager ability, leadership and support of nurses; and staffing and resource adequacy. Most studies in SA among RNs and CSN’s highlight dissatisfaction with staffing and resources [25, 26], but this study additionally highlights dissatisfaction with nurse participation in hospital affairs and nurse manager’s ability, leadership and support of nurses. It is well established that involving nurses in hospital governance and decision making improves patient outcomes, namely rating of the hospital, recommendation of the hospital [19], nurse-reported quality of care and patient safety climate [19, 27, 39, 44], patient mortality and patient satisfaction [12]. However, authors agree that not only should nurses participate in governance and decision making, but their contributions must be accepted and appreciated, as this allows them to feel empowered and become self-actualised [27, 45]. Similarly, nurse manager’s ability, leadership and support of nurses is linked to positive patient outcomes, including higher patient satisfaction and quality of care, and lower patient mortality, less adverse events, restraint use, complications of immobility, fractures, medication errors, patient falls, catheter use, pressure ulcers, inadequate pain management, hospital-acquired infections, and decreased length of hospital stay [46, 47] ENAs viewed nurse manager ability, leadership and support of nurses, and staffing and resource adequacy, more positively than any other category of staff, while CSNs experienced the subscale of resource adequacy more negatively than any other category of staff. Holtzhausen et al. [26]. also found CSNs to be most dissatisfied with this subscale in their study.

Regarding nurse perceptions of quality of care, nurses were somewhat confident that management will act to resolve problems in patient care that nurses identify, and that patients and their caregivers could manage their care after discharge. With regard to perceptions of patient safety, nursing staff felt neutral regarding communication errors, relying on temporary staff, that their mistakes were held against them and a lack of support for staff involved in patient safety errors. In all aspects, CSNs rated quality of care and patient safety more negatively, followed by RNs and ENs, while ENAs always rated the aspect most positively. Few studies could be found that made direct comparisons between different categories of nurses. Regionally, Alhassan et al. [30]. found that RNs had a greater knowledge and awareness of adverse medical events than the ENAs did. In SA, Swart et al. [32]. found that ENs in the Gauteng Province rated quality of care delivered to patients, overall grade of safety, that actions are taken to prevent errors from happening and that important information is lost during shift changes or when transferring patients from one unit to the next, more positively than RNs. However, they did experience that mistakes were held against them more than the RNs did. This would be similar to the conclusions of Truxillo et al. [29] that the more education the nurse has, the more competently they are able to assess quality of care and patient safety. CSNs, with little clinical experience, have a theory-practice gap, as research shows that there is little application of patient safety knowledge in new graduates, as they tend to prioritise care in a linear fashion working according to lists or guidelines, rather than having a holistic perception of patient care [48].

Nurse (all categories) perceptions of quality of care and patient safety were most correlated with the subscale of nurse foundations of quality of care and nurse manager ability, leadership and support of nurses. Nurses’ perceptions of quality of care focuses on patient care that is provided based on a nursing model rather than a medical model, where the nurse as a primary caregiver makes use of nursing diagnoses and provides nursing care through daily, up-to-date care plans for patients and continuity of care. The nursing care model further aims to improve quality of care through active staff development and continuing education programmes and preceptor programmes. A systematic review showed that nursing care models are linked to improved nurse outcomes, especially with regard to job control and autonomy, and patient outcomes [49]. Several systematic reviews looking at aspects of life-long learning, that include post-basic education, staff development programmes and continuing education programmes, state that continuous learning improves nurses’ knowledge, skills, attitudes and self-efficacy, as well as patient outcomes, patient care standards and cost [50–52].

Several systematic reviews provide evidence that nurse manager ability, leadership and support of nurses not only impacts on patient outcomes including patient satisfaction and quality of care, patient mortality, adverse events, restraint use, complications of immobility, fractures, medication errors, patient falls, catheter use, pressure ulcers, inadequate pain management, hospital-acquired infections, and decreased length of hospital stay [46, 47], but also on the development of a positive practice environment [53]. Nurse managers’ experience and advanced education have also been found to have a direct positive effect on quality of care and patient safety [54], while nurse managers exhibiting toxic behaviours resulted in increased frequency of nurse-reported adverse events and poorer quality of care [55]. It is therefore disconcerting to note that this aspect was considered as unfavourable - since it plays the most critical role in the improvement of quality of care and patient safety.

Adverse events affecting patients were most correlated with the collegial nurse-physician relationship subscale, while adverse events impacting nurses were most correlated with the foundations of quality of care subscale. Smith et al. [56]. mention that adverse events are most commonly linked to communication errors and shortage of staff, both of which have been highlighted by nurses in this study. However, in this study nurses specifically felt that the nurse-physician relationships were correlated with adverse events affecting patients, which was also highlighted by the systematic review of Norful et al. [57]. that linked nurse-physician teamwork and co-management with alleviating individual workload, preventing burnout, improving patient care quality, and leading to increased patient access to care. The subscale of nurse foundations of quality of care was also linked to adverse events impacting nurses, which is of course related to the fact that nursing care models are linked to more job control and autonomy; as well as educational interventions which ensure that staff are clinically competent.

Griffiths et al. [58]. suggested that targeted interventions to improve any of the practice environment subscales will lead to improved quality of care, which will automatically improve nurse outcomes. Therefore, it is recommended that more focus is placed on the two subscales of the practice environment that had the most impact on quality of care and patient safety, namely foundations of quality of care, and nurse manager ability, leadership and support of nurses – especially the latter, that was rated as unfavourable by nurses. It is recommended that targeted educational interventions be implemented for leadership development, as this is considered one of the most effective methods of improving this aspect [59].

The limitations of the study include its reliance on cross-sectional data, which limits the ability to assert causality between the perceptions of nurses with regard to their practice environment and the patients’ outcomes. Owing to the collection of data by using self-report, paper-based surveys, the study may have limited the narrative view of the participants regarding the phenomenon of the practice environment of nurses and patient oucomes. Furthermore, the sampling of hospitals in the province was purposively selected, and although it included a large set of participants, it is not a probability sample; therefore, the findings are not generalisable to other provinces of SA. The CSN population was also very small, and therefore the findings from this group should be interpreted with caution.

## Conclusion

Improving the practice environment, especially with regard to the subscale of nurse foundations of quality of care and nurse manager ability, leadership and support of nurses, is associated with improved quality of care. Nurses with higher qualifications, RNs and CSNs, rated quality of care lower than other categories of nurses did, contributing to the literature that higher-qualified staff are more competent to assess the practice environment and quality of care.

## Data Availability

The datasets analysed during the current study are available from the corresponding author on reasonable request.

## Abbreviations

AHRQ: Agency for Healthcare Research and Qualiity
CSN: Community Service Nurse
DoH: Department of Health
EN: Enrolled nurse
ENA: Enrolled nurse auxiliary
NWP: North West Province
RN: Registered nurse
SOPS: Surveys on patient safety
